# The Expression of T Cell FOXP3 and T-Bet Is Upregulated in Severe but Not Euthyroid Hashimoto's Thyroiditis

**DOI:** 10.1155/2016/3687420

**Published:** 2016-07-05

**Authors:** Stana Tokić, Mario Štefanić, Ljubica Glavaš-Obrovac, Sonja Jaman, Eva Novosadová, Jana Petrkova, Zdenka Navratilova, Mirjana Suver Stević, Martin Petrek

**Affiliations:** ^1^Department of Molecular Diagnostics and Tissue Typing, Osijek University Hospital, Josipa Huttlera 4, 31000 Osijek, Croatia; ^2^Faculty of Medicine, University of Osijek, Cara Hadrijana 10E, 31000 Osijek, Croatia; ^3^Laboratory of Immunogenomics, Department of Pathological Physiology, Faculty of Medicine and Dentistry, Palacký University, 775 20 Olomouc, Czech Republic; ^4^Clinical Institute of Nuclear Medicine and Radiation Protection, Osijek University Hospital, Josipa Huttlera 4, 31000 Osijek, Croatia

## Abstract

Hashimoto's thyroiditis (HT) is an organ-specific autoimmune disorder characterized by progressive thyroid failure. Th1 and Treg subset of CD4^+^ cells have been implicated in the pathogenesis; however, less is known about their respective roles across the spectrum of HT clinical presentations. To shed more light on CD4^+^ subsets role in HT, we investigated the mRNA expression levels of several Th1/Treg-associated transcription factors (T-bet/ETS1, HIF1*α*/BLIMP1/FOXP3) in peripheral blood T cells of 10 hypothyroid, untreated HT patients, 10 hypothyroid patients undergoing hormone replacement therapy, 12 euthyroid HT subjects, and 11 healthy controls by the qRT-PCR. Compared to euthyroid HT patients and controls, both hypothyroid (2.34-fold difference versus controls, *P* < 0.01) and thyroxine-supplemented patients (2.5-fold, *P* < 0.001) showed an increased FOXP3 mRNA expression in T cells. Similarly, mRNA expression levels of T-bet were upregulated in severely affected but not in euthyroid HT subjects (2.37-fold and 3.2-fold, hypothyroid and thyroxine-supplemented HT patients versus controls, resp., *P* < 0.01). By contrast, no differences in mRNA expression levels of ETS1, BLIMP1, and HIF1*α* were observed across the study groups. In summary, severe but not euthyroid HT was associated with robust upregulation of T-bet and FOXP3 mRNA in peripheral T cells, independent of the thyroid hormone status but proportional to disease activity.

## 1. Introduction

Hashimoto's thyroiditis (HT) is the second most widespread endocrine disorder characterized by autoimmune thyroid destruction and subsequently hypothyroidism. Traditionally, the pathogenesis of HT has been characterized by thyroid homing of autoreactive T-helpers 1 (Th1), cytotoxic T-lymphocytes, and NK cells, all of which are involved in targeted killing of thyroid follicular cells through antibody-mediated cytotoxicity and apoptosis [1].

Recently, two new subsets of Th cells, Th17, and regulatory T (Treg) cells have been implicated in HT pathogenesis [2–8]. Consequently, the imbalance between Th1, Th17, and CD4^+^CD25^+^Treg cells has been associated with thyroid inflammation [3–8], disease severity [6–8], and HT destructiveness [3, 8].

Th1 and Treg cell polarization is driven by lineage-specific master regulators, encompassing transcription factors (TFs) such as T-bet and FOXP3. In addition, several noncanonical TFs have been recently recognised to affect differentiation and function of Th1/Treg cells. Among these, ETS1 is essential for mounting effective Th1 responses [9, 10] and maintenance of Treg suppressive function by upregulating FOXP3 [11]. By contrast, the transcriptional repressor BLIMP1 (encoded by the PRDM1 gene) is predominantly involved in attenuation of proinflammatory Th1 and Th17 responses through negative regulation of INF-*γ*, T-bet, and interleukin- (IL-) 2 [12, 13]. In CD4^+^Tregs, BLIMP1 is highly expressed and supports Treg suppressive function by upregulating IL-10 [14]. Finally, a novel Treg/Th17 TF, hypoxia inducible factor (HIF)1*α*, has been proven essential factor for Th17 differentiation by skewing Treg polarization through FOXP3 destabilization and cooperative action with ROR*γ*t [15]. However, the importance of these factors for the pathogenesis of HTs has been rarely addressed; moreover, most of the studies have considered HT singular phenotype regardless of the disease complexity, particularly given the differences in thyroid function across the clinical spectrum of disease.

To address the role of T cell TFs in HT, we determined Th1 (T-bet, ETS1) and Treg-associated (HIF1*α*, BLIMP1, and FOXP3) mRNAs in peripheral blood T cells of HT patients. To explore possible differential expression at different clinical endpoints, we performed subanalyses in disease subsets characterized by distinct stage and residual thyroid function.

## 2. Materials and Methods

### 2.1. Subjects

Blood samples from healthy volunteers and patients were obtained at the Clinical Institute of Nuclear Medicine and Radiation Protection, Osijek University Hospital, Osijek, Croatia. HT was diagnosed as previously described [16] and 32 patients were age-matched, sex-matched, and classified into three subgroups according to the thyroid hormone levels at the time of diagnosis. The study comprised (1) 12 spontaneously euthyroid HT patients (EU HT), (2) 10 hypothyroid, untreated patients (HYPO HT), and (3) 10 hypothyroid HT cases rendered euthyroid by thyroxine (L-T4) replacement therapy [HYPO SUBST, mean L-T4 dose 1.13 *μ*g/kg body weight daily, median pretreatment serum thyrotropin (TSH) 15.1 mU/L, and interquartile range 11.5–35.3 mU/L]. The median follow-up for the entire patient cohort was 7 (interquartile range 5–8) years.

The control group (CTRL) consisted of 11 randomly selected, healthy, euthyroid, thyroid peroxidase autoantibodies- (TPOAb-) negative individuals, with no evidence or family history of autoimmune and endocrine disorders. All control subjects had normal ultrasound findings of thyroid gland. All participants were unrelated adults from eastern Croatia. Informed consent in written form was obtained prior to the study and study protocol was reviewed and approved by the institutional ethical committee.

### 2.2. Thyroid Function Measurement

TSH (normal range: 0.46–4.7 mIU/L, Vitros TSH Reagent Pack), free triiodothyronine (FT3) (1.9–5.7 pmol/L, Vitros FT3 Reagent Pack), and free T4 (FT4, 10–22 pmol/L, Vitros FT4 Reagent Pack, all from Ortho-Clinical Diagnostics, Amersham, UK) were measured in morning serum samples, taken between 8 a.m. and 12 p.m., according to the manufacturer's instructions. Maximum pretreatment serum TPOAb-IgG (50–125 kIU/L) was measured by ELISA (Anti-TPO Kit, Milenia Biotec, Germany) calibrated against WHO reference MRC 66/387a. Hypothyroidism was defined as clinically significant when TSH was >4.7 mU/L and FT4 was <10 pmol/L, but it was considered subclinical or latent if TSH was >4.7 mU/L and FT4 > 10 pmol/L.

### 2.3. Peripheral Blood Mononuclear Cells (PBMC) Isolation

PBMC were isolated from peripheral blood by density gradient centrifugation on Lymphoprep (Axis Shield, Oslo, Norway) as described by Böyum [17]. Briefly, heparinized blood samples were diluted 1 : 1 with 0.9% NaCl and following application to the Lymphoprep centrifuged for 20 min at 1000 ×g. Fraction of total lymphocytes and monocytes was harvested and rinsed with saline and then pelleted by centrifugation for 10 min at 550 ×g. The procedure was repeated twice. Cells were resuspended in 1 mL of the isolation buffer (PBS without Ca^2+^ and Mg^2+^ with 0.1% BSA and 2 mM EDTA) and counted after trypan-blue staining using the Bürker-Türk counting chambers.

### 2.4. Lymphocyte Subsets Separation

T-lymphocytes were separated from mononuclear cells by negative selection using untouched human T cells isolation kit, magnetic beads (depletion Dynabeads), DynaMag*™* magnet, and antibody mix (Invitrogen, Paisley, UK). Briefly, PBMC (1 × 10^7^) were incubated for 20 min at 2–8°C with heat inactivated fetal bovine serum and mouse monoclonal antibodies specific for CD14, CD16, CD19, CD36, CD56, CDw123, and CD235. After the incubation, cells were washed with the isolation buffer and pelleted by centrifugation at 300 ×g for 8 min. The pellet was resuspended and incubated for 15 min with prewashed magnetic beads, coated with monoclonal human anti-mouse IgG antibody. The bead-bound cells were subsequently separated on a DynaMag magnet, leaving cell suspension free from B-lymphocytes, NK cells, monocytes, platelets, dendritic cells, granulocytes, and erythrocytes. The remaining untouched CD4^+^ and CD8^+^ T-lymphocytes were transferred to a new tube. Steps involving washing and binding of bead-bound cells to a magnet were repeated twice. Residual Dynabeads were removed by placing the tube in a magnet for 2 min and then transferring the supernatant to a new tube.

### 2.5. Total RNA Extraction

Total RNA was extracted from untouched T-lymphocytes using TRI REAGENT (Sigma, USA) solution as described by Chomczynski and Sacchi [18]. Quality of total RNA was checked by ethidium bromide staining in 2% agarose gel electrophoresis and OD_260_/OD_280_ ratio > 1.8 measurements by NanoDrop 1000 spectrophotometer (Thermo Fisher Scientific, USA). Out of 43 samples of total RNA, 9 samples (20.9%) suspected for DNA contamination underwent DNase treatment using the Ambion® DNA-free*™* DNA Removal kit (Life Technologies, Carlsbad, USA) according to the manufacturer's instructions.

### 2.6. cDNA Synthesis

cDNA synthesis was performed using Transcriptor First Strand cDNA Synthesis Kit (Roche, Mannheim, Germany). For better coverage of the transcriptome, both anchored oligo-dT (2.5 *μ*M) and random hexamer primers (60 *μ*M) were added to 40 *μ*L master mix comprising 400 ng of total RNA, dNTP mix (1 mM each), 40 U of RNase inhibitor, and 20 U of reverse transcriptase. The run protocol was set at 65°C for 10 min for initial denaturation of RNA/primer mix and, after adding cDNA synthesis mix, continued at 25°C for 10 min, 55°C for 60 min, and 85°C for 5 min. All cDNA samples were diluted 8-fold to 1.25 ng/*μ*L end-point concentrations and stored in aliquots at −20°C until use.

### 2.7. Quantitative Real-Time PCR

The mRNA expression levels of five target (FOXP3, ETS1, TBX21, BLIMP1, and HIF1*α*) and three reference genes (TBP, HPRT1, and RPLPO) were measured by quantitative real-time polymerase chain reaction (qRT-PCR). The amplification of investigated transcripts was performed with the Rotor Gene 3000 instrument (Corbett Research, USA) in a 15 *μ*L total volume, containing 6.75 *μ*L of cDNA, 7.5 *μ*L of TaqMan Universal PCR Master Mix II kit, and 0.75 *μ*L of predeveloped individual TaqMan gene expression assay (Applied Biosystems). The cycling conditions were set according to the guidelines in the manufacturer's leaflet. The list of assays and amplicon sizes of the investigated genes is given in Table 1.

All qRT-PCR reactions were performed in triplicate and no-template negative control was included in each experiment. The reverse transcription negative controls without RT enzyme or template cDNA were run in parallel and did not yield any PCR product. At the end of each PCR run, real-time fluorescence measurements were collected at threshold cycle (Ct) defined as the fractional cycle number at which fluorescence passes fixed threshold of 0.05. Real-time PCR result was called an outlier at SD (Ct) > 0.3 and accordingly 9 (0.7%) out of 1278 PCR reactions were excluded from further analyses. Intra-assay variability was less than 0.92% for all investigated transcripts and less than 1.21% between different PCR experiments. All data were exported to GenEx software, and NormFinder and geNorm algorithms were used for identification of the most stably expressed reference gene. Pairwise analysis performed in geNorm identified TBP and HPRT1 as the best gene pair nominated by identical values of stability measure (M = 0.4922). TBP was selected as the most stable gene showing the lowest intra- (SD ± 0.04) and intergroup (SD ± 0.12) variability. Finally, fold difference in mRNA levels in unknown samples versus controls was calculated in comparison to TBP endogenous control using efficiency corrected model of ΔΔCt relative quantification method as described by Pfaffl 2001 [19].

### 2.8. Statistical Analysis

Data are presented as arithmetic means ± standard deviations (SD) or medians with interquartile ranges (IQR) where applicable. Kruskal-Wallis test with Bonferroni-modified Dunn's post hoc analysis was used for group comparisons. Pairwise correlations were determined by Spearman rank test. Two-tailed *P* < 0.05 was considered significant. If not otherwise stated, all statistical analyses were performed with NCSS2007 v07.1.20, NCSS LLC., Kaysville, USA.

## 3. Results

### 3.1. Subject Characteristics

Biochemical and demographical data are presented in Table 2. No differences in sex composition were found among the groups and no difference in serum FT3 and TSH values was seen between healthy controls and euthyroid and L-T4 substituted HT patients. Generally, higher FT4 levels were required to match the FT3 values in controls, most likely reflecting both morning intake of L-T4 shortly before blood collection and a trend for lower FT3 in L-T4 treated HT patients. As expected, FT4 serum levels were reduced and TSH levels significantly increased in hypothyroid patients when compared to the controls and L-T4 substituted subjects.

### 3.2. Expression Profiles of Investigated Th1-Associated Transcription Factors

**Figure 1:**
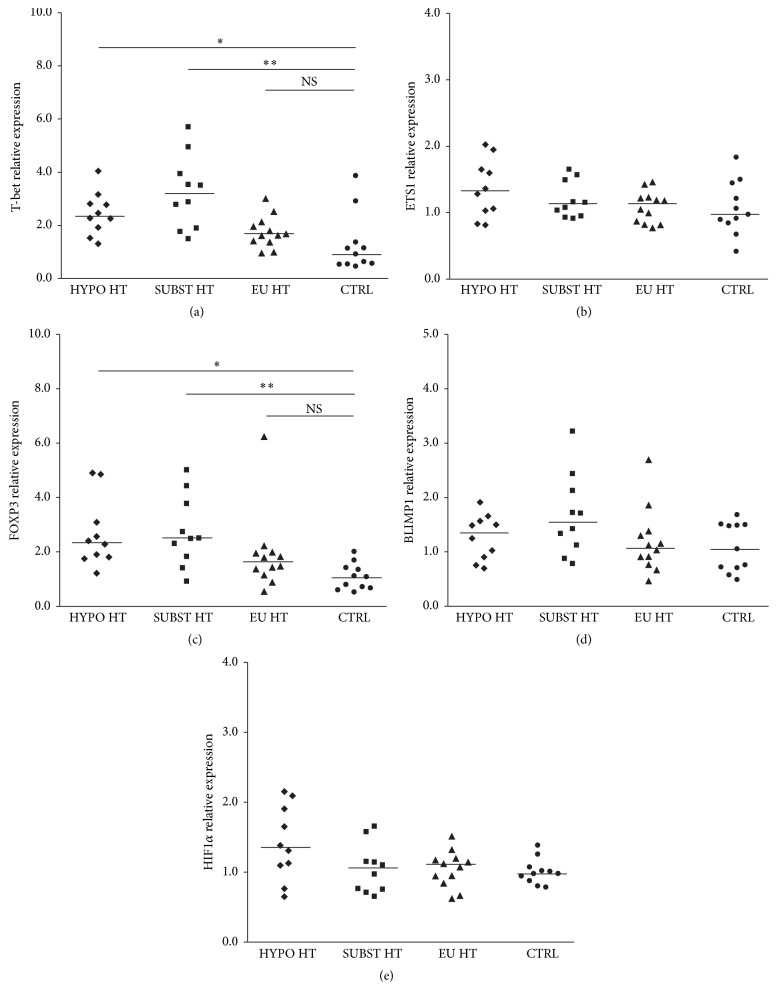
Relative mRNA levels of Th1/Treg-associated TF in HT patients and healthy controls. Compared to healthy controls (CTRL), (a) T-bet and (c) FOXP3 mRNA levels were upregulated in hypothyroid, both untreated (HYPO HT, ^*∗*^ *P* < 0.01, Kruskal-Wallis test and Bonferroni-Dunn's post hoc comparison) and thyroxine-supplemented (SUBST HT, ^*∗∗*^ *P* < 0.001) patients but not in spontaneously euthyroid HT (EU HT) subjects. Conversely, differences in T-bet and FOXP3 mRNA levels, compared between HYPO HT and SUBST HT or between EU HT and CTRL subgroups, were not significant (*P* > 0.05 for all). Similarly, no difference was found in (b) ETS1, (d) BLIMP1, or (e) HIF1*α* mRNAs levels across the studied groups.

Regarding expression of Th1 regulators, T-bet was upregulated in peripheral T cells isolated from hypothyroid, both untreated [median fold change 2.37, IQR (2.0–2.8), and *P* < 0.01] and thyroxine-supplemented patients [3.2-fold (2.13–3.85), *P* < 0.001], but not in spontaneously euthyroid HT group [1.66-fold (1.4–2.0), *P* > 0.05] or healthy controls [0.93-fold (0.56–1.26)] (Figure 1(a)). No difference in T-bet mRNA levels was observed between the treated and untreated hypothyroid patients with severe HT. Similarly, no significant difference was observed between euthyroid HT patients and healthy controls. Further, ETS1 mRNA levels were not different when compared across HT stages [HYPO HT, SUBST HT, and EU HT; 1.32 (1.04–1.64), 1.12 (0.97–1.41), and 1.12 (0.86–1.22), resp., *P* > 0.05 for all] or healthy controls [0.97 (0.87–1.33)] (Figure 1(b)).

### 3.3. Expression Profiles of Treg-Associated Regulators

Concerning Treg regulatory molecules, FOXP3 mRNA levels closely mirrored T-bet expression in untreated and thyroxine-supplemented patients (global *P* = 6.03 × 10^−4^), showing 2.34-fold (1.83–2.96) and 2.5-fold (1.95–3.52) increment in median expression values when compared to the healthy controls, respectively (Figure 1(c)). No difference between hypothyroid patients, treated and untreated (2.34-fold versus 2.5-fold, *P* > 0.05), or between spontaneously euthyroid HT subjects and controls was observed (1.63-fold versus 1.09-fold, *P* > 0.05). No changes across the studied groups [HYPO HT, SUBST HT, EU HT, and CTRL] were observed when BLIMP1 [1.37 (0.93–1.55); 1.57 (1.18–2.03); 1.08 (0.87–1.32); 1.06 (0.72–1.49), resp., *P* > 0.05 for all, Figure 1(d)] or HIF1*α* transcripts were measured [1.35 (1.1–1.84); 1.04 (0.76–1.15); 1.09 (0.92–1.18); 0.98 (0.91–1.05), resp., *P* > 0.05 for all, Figure 1(e)].

### 3.4. Correlation Analysis

**Figure 2:**
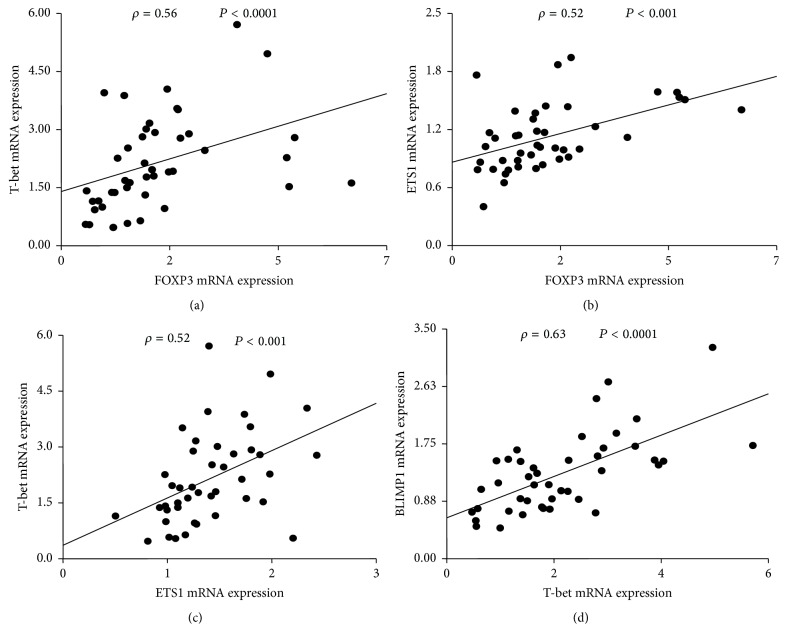
Spearman pairwise correlation analysis of T-bet, ETS1, FOXP3, BLIMP1, and HIF1*α* mRNA levels in pooled samples (*n* = 43). FOXP3 positively correlated with (a) T-bet and (b) ETS1. T-bet was related to (c) ETS1 and (d) BLIMP1. *ρ*: Spearman correlation coefficient; global significance *P* < 0.05.

To examine if there is any relationship between mRNA levels of Th1/Treg cell-associated regulators in peripheral T cells, we performed pairwise correlation analysis on pooled samples. A number of significant relationships were observed, as revealed by Spearman rank test (Figure 2).

Accordingly, elevated FOXP3 mRNA levels consistently coincided with mRNA levels of T-bet (Spearman's rho, *ρ* = 0.56, *P* = 0.0001, Figure 2(a)) and ETS1 (*ρ* = 0.52, *P* < 0.001, Figure 2(b)). In line, T-bet and ETS1 were also positively related (*ρ* = 0.52, *P* < 0.001, Figure 2(c)). Additionally, increased T-bet expression was positively and independently correlated with BLIMP1 mRNA abundance (*ρ* = 0.63, *P* < 0.0001, Figure 2(d)).

A possible association between mRNA expression profile, age, and thyroid hormone levels was also investigated. Among the studied genes, FOXP3 mRNA levels were negatively associated with FT3 (*ρ* = −0.44, *P* = 0.0035) whereas ETS1 levels were weakly, albeit significantly negatively, correlated with FT4 levels (*ρ* = −0.35, *P* = 0.027). Additionally, a positive relationship was observed between age and BLIMP1 mRNA levels (*ρ* = 0.45, *P* = 0.0024). After correction for multiple comparisons (Bonferroni corrected *P* < 0.0018), these relationships did not attain significance (data not shown).

## 4. Discussion

In the present study, increased mRNA expression of T-bet, a master regulator of the Th1 lineage, was observed in severely affected, hypothyroid patients, both treated and untreated, but not in spontaneously euthyroid HT counterparts; in addition, the prototypical Treg FOXP3 message closely mirrored T-bet expression patterns in both HT stages. Taken together, the findings suggest a simultaneous deregulation of T-bet^+^ Th1/CD8^+^ and FOXP3^+^ Treg cell compartments in destructive, full blown HT, which seems to be less pronounced or absent in milder, euthyroid forms of the disease. Thus, distinct mechanisms may be engaged to different extent across the spectrum of thyroid autoimmunity, providing a solid basis for different therapeutical targets in HT.

Thyroid demission in HT is a multistep process, probably involving an individually variable set of mediators before a measurable effect develops. Th1 cells are critical T effectors (Teff) in the pathogenesis of spontaneous autoimmune thyroiditis in iodine-treated NOD-H2h4 mice [2, 20], and the proportion of peripheral Th1 cells is higher in patients with severe HT than in patients with mild HT [6]. T-bet is a direct transcriptional regulator of Th1 cytokines [21], commonly prevalent in both thyroid and PBMC of HT patients [22], and previously related to high TPOAb titres [23], disruption of thyroxisomes [24, 25] and apoptosis of thyroid follicular cells during in vitro induction of destructive autoimmune thyroiditis [26]. Accordingly, development of more destructive disease forms in HT seems to be driven by Th1 upregulation, and indeed we have recorded disease stage-dependent increment in T-bet mRNA levels in patients with severe HT. In contrast, FOXP3^+^Treg lymphocytes play a nonredundant role in the protection against thyroid autoimmunity, as Treg depletion was shown to induce a Th1-dependent form of EAT in mice [20], while diminished Treg cell numbers and attenuated FOXP3 gene expression were found in PBMC of HT patients [7, 8]. Nevertheless, the existence of a functional rather than “numerical” deficiency in Treg cells has also been suggested in HT. In a single AITD study, a nonspecific defect in Treg regulatory function was observed in vitro, despite increased numbers of FOXP3^+^CD69^+^CD4^+^ T cells in both thyroid and PBMC of patients with AITD [27]. In yet another study, an elevated baseline production of mRNA encoding FOXP3Δ2, an exon 2 splicing variant central to the glycolytic control of suppressive Tregs [28], was observed [29].

Despite the peripheral enrichment of FOXP3^+^ T cells in our severe HT patients, thyroid inflammation is obviously still not suppressed, thus supporting the concept of a profound insufficiency of CD4^+^FOXP3^+^ Treg cells in autoimmunity, previously also reported in rheumatoid arthritis [30], inflammatory bowel disease [31], and Graves' disease [27]. Underlying molecular mechanisms are currently controversially discussed: Treg suppressive capacity might be diminished by ligation of Treg expressed GITR (glucocorticoid-induced tumor necrosis factor receptor) by GITRL found in serum and inflamed thyroid tissue [7, 27]; once in the target organ intrathyroidal Tregs might suffer from apoptosis-induced decrease [32], or responder T effector cells may show resistance to Treg-mediated suppression due to the predominance of proinflammatory cytokines in the microenvironment, a mechanism previously demonstrated in vitro [33] and in vivo [34].

Alternatively, of interest is the possible transition of Treg lymphocytes towards proinflammatory Th17 cells, a phenomenon that may further contribute to the perpetuation of the autoimmune process. Indeed, Tregs may rapidly transdifferentiate towards Th17 phenotype [35] when exposed to cytokine milieu enriched with IL-6, IL-17, and IL-23 [36]. Such Th17 differentiation cues were previously reported in serum and thyroid tissue of HT patients [3–5, 22] and associated with loss of Teff/Treg cell balance and progression towards severe hypothyroidism [7, 8].

Finally, there is accumulating evidence that CD4^+^CD25^+^FOXP3^+^Treg cells are heterogeneous in their commitment stages and expression of effector molecules of suppression [37]. This functional diversity of Treg cells is driven by specific TFs distinct from FOXP3, which coordinate a phenotypic and functional specialization of Th and Treg cells in parallel during different types of inflammatory response. Among these, T-bet is expressed by a subset of Treg cells [38] and is required for Tregs to suppress Th1 inflammatory responses [39]. Consequently, a proportionate relationship of FOXP3^+^Treg cell frequency with generalized T cell activation and Th1 magnitude has been observed in vivo [40]. Thus, experimental differences aside, Treg homeostasis may be far more complex in AITD than currently appreciated and further work is required for understanding how different Treg subsets and their diverse functional properties are controlled in vivo [41].

In activated Tregs, evolutionarily conserved noncoding sequences located in the FOXP3 intronic regions, CNS2, help protect FOXP3 expression from destabilizing cytokine conditions [42] in an inflammatory environment. A number of TFs, including, but not limited to, ETS1 and FOXP3 itself can interact with CNS2, whereas the expression of other TFs such as T-bet [38], BLIMP1 [14], and HIF1*α* in Tregs can also affect specific aspects of Treg functions [15]. Among these, HIF1*α* suppresses Treg development by binding FOXP3 and targeting it for proteasomal degradation [15]; T cell-specific BLIMP1 deficiency exacerbates autoimmune pathology by increasing the ratio of Teff (Th1 and Th17) to Tregs [43], while ETS1 deficiency severely impairs the differentiation and function of both Th1 and Treg, but not Th17 cells [9, 11, 44]. A number of putative correlations observed between TFs in our study are broadly consistent with these presented knockout data.

Nevertheless, a question remains about the exact source of these mRNAs. T-bet is broadly, albeit not exclusively, expressed in human CD4^+^Th1, cytotoxic, and memory CD8^+^ T cells. FOXP3 is the master regulator of the CD4^+^ Treg gene expression program, playing also a less clear role in CD8^+^FOXP3^+^ Treg cells. Moreover, ETS1, BLIMP1, and HIF1*α* TFs are differentially engaged in multiple signalling pathways in CD4^+^ and CD8^+^ committed T cells and are alternatively expressed with respect to cell lineage, developmental stage, and function [9, 15, 43–46], all of which may be responsible for observed mRNA data. Although no decisive role could be ascribed under the current experimental conditions, the limitations of the current study warrant further research into their roles in HT. Hence, several restrictions should be emphasized. (I) No cell sorting was performed and no protein level data were obtained. (II) Western blot should be used for analysis of HIF1*α* protein levels. (III) The precise contribution in CD4^+^ versus CD8^+^ T cells or in naïve versus Teff cells was not investigated. No functional studies of different T cell subsets were performed and little information from affected tissues is available. The study could be significantly improved by both the inclusion of thyroid tissue samples and the analysis of expression of additional Th17/Treg cytokines and master regulators. (IV) The relatively small sample size is an additional limitation to optimal sensitivity; thus, the cohort was exploratory in nature. Nevertheless, strict selection criteria were applied, resulting in a well-characterized cohort regarding demographics, treatment exposures, thyroid residual function, and disease status. This stratification is crucial in pursuit for pathogenetic components in HT and may be an explanation for contradictory results in several similarly sized but phenotypically mixed studies. Finally, majority of patients in this study were female whites of European ancestry, thus limiting additional effects of sex or ethnicity.

In conclusion, our data provide preliminary evidence that change from mild to severe HT types is encouraged by parallel alterations in T-bet^+^ and FOXP3^+^ T cell compartments, independent of reductions in thyroid hormone levels, but proportional to disease stage. Even though underlying mechanisms remain beyond the competence of this study, our findings warrant further research into FOXP3^+^T-bet^+^ T cell compartment in HT and may contain relevant investigation targets that will accelerate our understanding of Treg insufficiency in autoimmunity. However, further work is necessary for accurate dissection of the functional and numerical differences between FOXP3^+^ and T-bet^+^ T cell subsets of HT patients observed in this study.

## Figures and Tables

**Table 1:** List of TaqMan probes and amplicon sizes of investigated genes.

Assay ID	Gene abbreviation	Gene name	GenBank accession number	Function	Amplicon size (bp)
Hs00428293_m1	ETS1	Erythroblastosis virus E26 oncogene homolog 1 (avian)	NM_001143820.1	Transcription factor, involved in mounting of Th1 response and inhibition of Th17 development	99

Hs01085834_m1	FOXP3	Forkhead box P3	NM_014009.3	Transcriptional regulator of Treg cell development and function	61

Hs00153153_m1	HIF1A	Hypoxia inducible factor 1	NM_001530.3	Transcriptional regulator, activator of ROR-*γ*t expression and Th17 differentiation	76

Hs99999909_m1	HPRT1	Hypoxanthine phosphoribosyltransferase 1	NM_000194.2	Purine metabolism	100

Hs00153357_m1	PRDM1/BLIMP1	PR domain containing 1, with ZNF domain/B-lymphocyte-induced maturation protein 1	NM_001198.3	Repressor of beta-interferon (*β*-IFN) gene expression	65

Hs00420895_gH	RPLP0	Ribosomal protein, large, P0	NM_001002.3	Ribosomal protein, component of 60S subunit	76

Hs00894392_m1	TBX21	T-box 21	NM_013351.1	T cell-specific T-box transcription factor	119

Hs99999910_m1	TPB	TATA box binding protein	NM_003194.4	Transcription factor	127

**Table 2:** Descriptive analysis of clinical and biochemical characteristics of patients and healthy controls.

	HYPO HT	SUBST HT	EU HT	CTRL
Subjects	10	10	12	11
Age	42.3 ± 4.62	57.7 ± 4.62	50.6 ± 4.22	45 ± 4.41
Gender (F/M)	8/2	9/1	12/0	10/1
FT4 (pmol/L)	11.01 ± 0.83	16.31 ± 0.83^*∗*^	12.12 ± 0.76	14.09 ± 0.8^*∗∗*^
FT3 (pmol/L)	2.91 ± 0.27	3.38 ± 0.27	3.24 ± 0.24	3.6 ± 0.25
TSH (mIU/L)	10.35 (5.25–13.1)	2.59 (0.9–3.0)^#^	3.11 (1.6–4.3)	1.63 (1.0–2.4)^##^
TPOAb (kIU/mL)	155 (61–2217)	246 (77–969)	677 (267–1112)	neg

Data are presented as mean ± SD or median with interquartile range (25th–75th percentile) according to the distribution. The study included 11 healthy controls (CTRL) and 32 HT patients classified as hypothyroid (HYPO HT), rendered euthyroid by thyroxine (L-T4) replacement therapy (SUBST HT), and spontaneously euthyroid (EU HT).

F: female and M: male. The *P* value represents different groups compared with hypothyroid HT patients.

^*∗*^ *P* = 0.00006; ^*∗∗*^ *P* = 0.01; ^#^ *P* = 0.1 × 10^−5^; ^##^ *P* = 0.1 × 10^−6^ (Kruskal-Wallis test and Bonferroni-Dunn's post hoc comparison).

FT4: free thyroxine (normal range: 10–22 pmol/L); FT3: free triiodothyronine (1.9–5.7 pmol/L); TSH: thyroid stimulating hormone (normal range: 0.46–4.7 mIU/L).
